# The role of JAK/STAT signaling pathway in cerebral ischemia-reperfusion injury and the therapeutic effect of traditional Chinese medicine: A narrative review

**DOI:** 10.1097/MD.0000000000035890

**Published:** 2023-11-17

**Authors:** Tianzhi Song, Yishu Zhang, Liangrong Zhu, Yuyan Zhang, Jingmei Song

**Affiliations:** a The Fourth School of Clinical Medicine, Zhejiang Chinese Medical University, Hangzhou, China; b The Second School of Clinical Medicine, Zhejiang Chinese Medical University, Hangzhou, China; c Wenling Hospital of Traditional Chinese Medicine, Taizhou, China; d School of Life Sciences, Zhejiang Chinese Medical University, Hangzhou, China; e School of Basic Medicine Sciences, Zhejiang Chinese Medical University, Hangzhou, China.

**Keywords:** apoptosis, cerebral ischemia reperfusion, JAK/STAT signaling pathway, nerve inflammation, traditional Chinese medicine

## Abstract

Cerebral ischemia is a cerebrovascular disease with symptoms caused by insufficient blood or oxygen supply to the brain. When blood supplied is restored after cerebral ischemia, secondary brain injury may occur, which is called cerebral ischemia-reperfusion injury (CIRI). In this process, the Janus kinase/signal transducer and activator of transcription (JAK/STAT) signaling pathway plays an important role. It mediates neuroinflammation and participates in the regulation of physiological activities, such as cell proliferation, differentiation, and apoptosis. After CIRI, M1 microglia is activated and recruited by the damaged tissue. The inflammatory factors are produced by M1 microglia through the JAK/STAT pathway, eventually leading to cell apoptosis. Meanwhile, the JAK2/STAT3 signaling pathway and the expression of lipocalin-2 and caspase-3 could increase. In the pathway, phosphorylated JAK2 and phosphorylated STAT3 function of 2 ways. They not only promote the proliferation of neurons, but also affect the differentiation direction of neural stem cells by further acting on the Notch signaling pathway. Recently, traditional Chinese medicine (TCM) is a key player in CIRI, through JAK2, STAT3, STAT1 and their phosphorylation. Therefore, the review focuses on the JAK/STAT signaling pathway and its relationship with CIRI as well as the influence of the TCM on this pathway. It is aimed at providing the basis for future clinical research on the molecular mechanism of TCM in the treatment of CIRI.

## 1. Introduction

Cerebral ischemia is cerebrovascular disease, with a series of symptoms producing, due to the lack of blood or oxygen supply to the brain. It is difficult to meet the need for metabolism. Cerebral hemorrhage accounts for about 80% of the deaths of cerebrovascular diseases, and its mortality rate is extremely high,^[1]^ leading to irreversible death of neurons.

Provided that blood circulation and oxygen supply are restored again after a certain period of ischemia, cell structure destruction and function in the ischemic area could be destroyed. This process is called cerebral ischemia-reperfusion injury (CIRI).^[2]^ It is the main reason that directly causes cerebral infarction and reduces the prognosis of patients with cerebral ischemia.^[3]^ Therefore, it is a subject in the field of cerebrovascular disease to find treatments to restore blood supply and reduce CIRI, and effectively protect cerebrovascular and brain tissue.

Neuroinflammation, apoptosis, cell proliferation and differentiation, are greatly hit in CIRI. Inflammation is one of the most important mechanisms leading to CIRI.^[4]^ The Janus kinase/signal transducer and activator of transcription (JAK/STAT) signaling pathway is an essential pathway inducing CIRI. It is primarily composed of the JAK family, STAT family, as well as their phosphorylation, p-JAK, and p-STAT. After cerebral ischemia, inflammatory factors or growth factors are secret, which combine with cytokine receptors on the JAK/STAT pathway. Then, JAK and STAT are phosphorylated to p-JAK and p-STAT. p-STAT combines with the target gene to up-regulate the expression of the downstream factors. The development of CIRI is determined by what kind of gene is expressed. Neuroinflammation is caused by inflammatory factors, like IL-6, and inflammatory cells, like astrocytes.^[5]^ Apoptosis is mainly related to the level of Bcl-2 or caspase-3, which are directly induced by STAT3.^[6]^ Cell proliferation and differentiation include the activation of neural stem cells or vascular endothelial cells.

Restoring the structure and function of damaged neurons are 2 main measures for the clinical treatment of CIRI. Nowadays, thrombolysis is widely used in Western medicine therapy.^[7]^ The recombinant tissue plasminogen activator is the only drug proven to be effective in the treatment of ischemic stroke.^[8]^ The drug protects neurons through the JAK/STAT pathway.^[9]^ However, its therapeutic window is narrow and has a high probability of complications, such as bleeding and damage to the blood-brain barrier.^[10]^ In contrast, TCM has little side effects, receiving much attention in recent years.

Based on dialectical thinking, traditional Chinese medical science has formed dialectical methods of disease diagnosis and treatment.^[11]^ Among them, “Qi deficiency and blood stasis” is one of the main pathogeneses.^[11]^ Correspondingly, activating blood circulation to remove blood stasis or invigorate qi and blood circulation is the primary method to treat stroke. Preliminary studies have shown that the TCM for blood circulation and stasis removal could not only resist oxidation, inhibit apoptosis, but also alleviate inflammatory reaction, which is conducive to improve CIRI.^[12]^ Meanwhile, the JAK/STAT pathway is an important target of the TCM. The TCM exerts anti-inflammatory effects mainly by down-regulating JAK2, STAT3, STAT1 and their phosphorylation.

In short, the influence of the JAK/STAT signaling pathway on CIRI is expounded in the review. Also, the TCM herbal compounds, pairs and ingredients for blood circulation and stasis removal that act on this pathway are summarized.

## 2. The structure and function of JAK/STAT signaling pathway

JAK/STAT pathway is primarily composed of extracellular signal molecules, tyrosine kinase-related receptors, JAK family, STAT family, their phosphorylation, p-JAK and p-STAT, as well as target genes. The following focus on the JAK, STAT, and how JAK/STAT pathway works.

### 2.1. The structure and function of JAK family

JAK constitutes the intracellular signal transduction part of cytokine receptors. JAK1, JAK 2, JAK 3 and TYK2 are included.^[13]^ JAK3 is expressed in hematopoietic cells, while other members are commonly expressed in tissues.^[14]^

**Figure 1.**
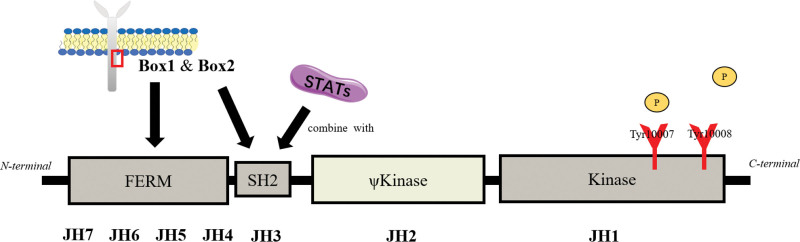
The structure of the JAK family. Box1 and Box2 present at tyrosine kinase-related receptors. JAK = Janus kinase.

Structurally, JAK has 7 JAK homology domains (JH) (Fig. 1). FERM, the N-terminal of JAK, is composed of JH5~7 and half of JH4, on which there is a Box1 binding site. The adjacent Src homologous region 2 (SH2) consists of JH3 and half of JH4. The intracellular binding site of STAT and Box2 on cytokine receptors are exposed to SH2.^[13,15]^ The downstream of SH2 is ψKinase and Kinase, which are respectively composed of JH2 and JH1. JH2 maintains the activity of JAK, while JH1 activates JAK by catalyzing the phosphorylation of tyrosine residues, including Tyr10007, Tyr1008.^[16]^

Functionally, JAK is closely related to cell proliferation, differentiation, and apoptosis. It mediates the signal transduction of growth hormone and hematopoietic factor.^[14]^ JAK performs an increasingly vital part in the occurrence and development of the inflammation, the autoimmune disease, and the myeloproliferative disease. Different JAK has similar functions, for instance, they are all found in the blood cancer. While different JAK often function specifically (Table 1).

**Table 1:** Summary of the function of JAK and STAT.

	Neural related applications	References
JAK1	Leading to auto-inflammation and have an impact on the response of IL. Decreasing apoptotic via the JAK1/STAT3/Bcl2 pathway, when injected of I interferon-β.	^[15,17,18]^
JAK2	Binding inflammatory factors, growth factor receptors. Promoting the proliferation of red blood cells and platelets.	^[16,19,20]^
JAK3	Regulating the expression of the creatine transporter CreaT in the brain. Promoting PI3-K-induced neuronal survival. Promoting proliferation of neuronal cell through microglia.	^[21–23]^
Tyk2	Influencing neurological function through neuronal cell death.	^[24]^
STAT1	Playing an role in value-added effect of the interferon-γ and promoting the expression of apoptosis-related genes. Facilitating cell proliferation and improves anti-inflammatory activity.	^[25–27]^
STAT2	Mediating interferon-α/β-mediated apoptosis and affecting neural stem cells. Regulating the shape of mitochondria to affect the viability of nerve cells.	
STAT3	Related to the regulation of cell growth, apoptosis, oxidative stress, neuroinflammation, immune regulation and autophagy. Involving in the neuroprotection of CIRI.	^[6,28,29]^
STAT4	Regulating Th1/Th2 differentiation and mitigating the neurotoxic effects produced by Th1. Increasing macrophages to affect inflammation.	^[30,31]^
STAT5	Reducing apoptosis mediated by erythropoietin or Bcl-xl, and conversely, causing changes in Bcl-2 and caspase-3 and mediating apoptosis. Influencing learning and memory abilities and regulating biological behavior.	^[32–36]^
STAT6	Promoting the proliferation of Th2 and increasing the expression of cytokines. Inducing the conversion of macrophages to M2 type, mediated by interleukin cytokines-4 and interleukin cytokines-13. Resulting in the inflammatory response and also the clearance of hematomas.	^[37–40]^

JAK = Janus kinase, STAT = signal transducer and activator of transcription, Th = helper T cell.

#### 2.1.1. The function of JAK1.

It has been proven that the tissue after JAK1 knock-out has an impact on the response of interleukin cytokines (IL), like IL-2, IL-4, IL-6 and IL-10. JAK1 is also associated with receptors of IL-2 and IL-6.^[15]^ It has been demonstrated that intranasal injection of interferon-β (IFN-β) could counteract apoptotic via the JAK1/STAT3/Bcl2 pathway.^[17]^ In the brain of mice with 22q11.2 deletion syndrome, activation of JAK1 leads to auto-inflammation and may cause neurological dysfunction.^[18]^

#### 2.1.2. The function of JAK2.

JAK2 could bind to the IFN-γ receptor, G protein-coupled receptor, vascular endothelial growth factor receptor and growth hormone receptor, and play a wide range of signal transduction roles.^[16]^

JAK2 is chiefly involved in epithelial cell differentiation, and inflammatory response. Knocking out JAK2-related genes may result in the loss of red blood cells, indicating that JAK2 is directly related to the production of red blood cells and platelets.^[19]^ As shown in the research, there is V617F in JAK2 ψKinase region, which is prone to mutation. The mutation probably leads to the myeloproliferative tumor.^[20]^

#### 2.1.3. The function of JAK3.

JAK3 is highly expressed in lymphocytes, relevant to immune function. The deficiency of JAK3 could cause the severe combined immunodeficiency syndrome, and dysfunction of lymphocytes. Additionally, JAK3 regulates the expression of the creatine transporter CreaT in the brain, causing intellectual disability with seizures.^[21]^ Increased expression of JAK3 mediates PI3-K-induced neuronal stockpiling after neural injury.^[22]^ JAK3-dependent activation of microglia modulates the proliferation and differentiation of neural progenitor cells, facilitating recovery from spinal cord injury.^[23]^

#### 2.1.4. The function of TYK2.

TYK2 is related to the cytoplasmic domain of type I, type II cytokine receptor and the type I, type III interferon receptor. TYK2, as a therapeutic target for autoimmune and inflammatory diseases, abnormally appears in respiratory diseases, such as asthma, chronic obstructive pulmonary disease, lung cancer, and cystic fibrosis.^[41]^ TYK2 also affects Alzheimer’s disease through neuronal cell death, indicating that it influences neurological function.^[24]^

### 2.2. The structure and function of STAT family

STAT is a kind of protein that exerts the biological effect by binding to target genes. STAT1, STAT2, STAT3, STAT4, STAT5a, STAT 5b, and STAT 6 are included.

**Figure 2.**
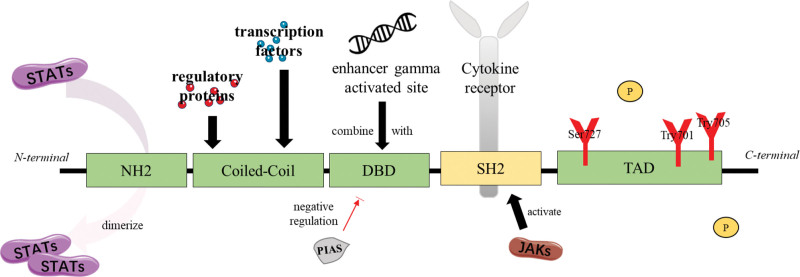
The structure of the STAT family. STAT = signal transducer and activator of transcription.

Structurally, STAT in mammals is divided into 6 functional segments from N-terminal to C-terminal, as shown in Figure 2. N-terminal domain2, Coiled-Coil domain, DNA binding domain, SH2, and transcription activation domain.^[42]^ N-terminal domain2 enables STAT homodimerize or heterodimerize. The coiled-Coil domain has a hydrophilic surface, which is probably combined with regulatory proteins and transcription factors.^[16]^ DNA binding domain is composed of β-immunoglobulin and could specifically bind to the enhancer gamma activated site. This process is negatively regulated by protein inhibitors of activated STAT (PIAS). The sequence of SH2 domain is highly conserved. It is the core part of STAT transcription regulation. Activated by JAK, the arginine residue of SH2 is the binding site of phosphorylated tyrosine on cytokine receptors, determining the specificity of binding cytokine.^[43,44]^ Transcription activation domain contains tyrosine and serine residues, which are phosphorylated by different proteins and responsible for the dimerization of STAT.^[6]^

Functionally, STAT widely participates in regulating signal transduction and gene transcription. Influenced by upstream factors, STAT is phosphorylated. Then, IL enters the nucleus and binds to specific sites in the target gene sequence, affecting the transcription level of DNA. Also, STAT act on other signaling pathways, affecting cell growth, differentiation, and apoptosis. It has been found that the stimulation and metastasis of STAT cause immune defense and cell proliferation.^[45]^

Besides, STAT family exists in many diseases, like tumor, CIRI, and the inflammatory bowel disease.^[46,47]^ Various STAT has a different regulatory role in signal transduction, some of which could act on the response of cytokines and regulate the activation of GF.^[28,48]^

#### 2.2.1. The function of STAT1.

Mediated by the IFN-γ, it is mainly related to the immune regulation of virus and cancers. STAT1 activation is necessary for the value-added effect of the IFN-γ.^[25]^ Meanwhile, the signaling pathway, where STAT1 locates, promotes the expression of apoptosis-related genes, such as caspases 2, caspases 3, caspases 7, then causing the apoptosis. Indeed, it also inhibits c-Myc to regulate the cell cycle.^[26]^ STAT1 also plays a major role in promoting cell proliferation and enhancing anti-inflammatory activity by targeting control genes.^[27]^

#### 2.2.2. The function of STAT2.

STAT2 exerts intranuclear regulation mainly in the form of STAT1-STAT2 heterodimer and affects the biochemical response of IFN-α/β.^[44]^ STAT2 is a key mediator in IFN-α/β-mediated apoptosis.^[49]^ In the absence of INF-α, STAT2 is activated, affecting neural stem cells.^[50]^ It has been reported that STAT2 regulates the shape of mitochondria, affecting the viability of neuronal cells.^[49]^ More research is still needed on the effect of STAT2 on neural injury.

#### 2.2.3. The function of STAT3.

Initially, STAT3 has been found in the release of IL-6, and it could promote the expression of genes in the acute phase. The experiment result has proved that STAT3 often forms p-STAT3 in hippocampus and corpus callosum.^[51]^ STAT3 is involved in the regulation of cell growth, apoptosis, oxidative stress, neuroinflammation, immune regulation and so on.^[28]^

Under the stimulation of ischemia and other factors, STAT3 in mitochondria gradually accumulates, causing autophagy.^[6]^

STAT3 is the target of TCM in current medicine and treatment research.^[14]^ Observing the activated opioid receptors after ischemia, researchers have found that STAT3 and p-STAT3 were involved in the neuro protection of CIRI.^[29]^

#### 2.2.4. The function of STAT4.

STAT4 is mainly expressed in lymphoid and myeloid tissues. STAT4 is mainly activated by IL-12, in addition to IFN-α/β and IL-23. STAT4 protein activation plays a key role in the regulation of helper T cell 1/helper T cell 2 (Th1/Th2) differentiation and the resulting dysregulation, leading to inflammatory diseases.^[30]^ In acute ischemic stroke, regulation of STAT4 gene levels alleviates the neurotoxic resulted by Th1.^[30]^

Also, studies have shown that transitory expression of STAT4 may increase macrophages and affect the prognosis of neurological tumors.^[31]^

#### 2.2.5. The typing and function of STAT5.

There are 2 subtypes of STAT5, including STAT5a and STAT 5b. Although they are transcribed by different genes, more than 90% of their structures are homologous.^[52]^ STAT5a is necessary for mammary gland development and milk production. STAT5b inhibits apoptosis of tumor cells by increasing Bcl-xl.^[32]^

The results concerning cerebral ischemia have pointed out that an enhanced phosphorylation of STAT5 exerts anti-apoptotic effect, mediated by erythropoietin.^[33]^ However, in contrast, it has been reported that STAT5 may downregulate Bcl-xl and activate caspase-3, promoting apoptosis.^[34]^ Additionally, STAT5 deficiency affects the learning ability and the memory, but not neurogenesis in the hippocampus.^[35]^ Besides, STAT5 could also affect brain function and regulate the maternal and feeding behavior.^[36]^

#### 2.2.6. The function of STAT6.

STAT6 is expressed in the mature brain.^[53]^ STAT6 could promote the proliferation of Th2,^[37]^ causing a decrease in the ratio of Th1/Th2. STAT6 also leads to an increase in the expression of cytokines such as IL-4 and IL-13, and promotes the gene transcription of IL-31.^[38]^ Meanwhile, the secretion of cytokines could increase and mediate the inflammatory response.

Unlike other STATs, induced by IL-4 and IL-13, STAT6 enable the macrophages converse to the M2 type, causing the inflammatory response in the brain.^[39]^ The process conversely promotes hematoma clearance and neurological recovery from cerebral hemorrhage.^[40]^

### 2.3. The regulatory process of JAK/STAT signaling pathway

#### 2.3.1. The activation of JAK/STAT signaling pathway.

The JAK/STAT pathway is a cytokine-activated signal transduction pathway.^[42]^ Firstly, extracellular signal molecules recognize and bind to the corresponding cytokine receptors. The extracellular signal molecules include inflammatory factors (e.g., IL-1, IL-6, IL-18, IFN-α/β, tumor necrosis factor-α),^[54]^ growth factors (e.g., erythropoietin, epidermal growth factor, platelet-derived growth factor) and growth hormone. Besides, oncogenes and boundary pile neurotrophic factors could also be served as extracellular signaling molecules. Cytokine receptors, also known as tyrosine kinase receptors, are transmembrane receptors with no kinase activity.^[16]^

Secondly, the subunits of the receptor tend to be oriented and oligomerized. There is a homologous region exists near the cell membrane on it, which binds to the functional region of JAK through Box1 and Box2. JAK is a non-transmembrane protein with selective specificity, indicating that different extracellular signaling factors could activate different JAK. One extracellular cytokine could bind multiple JAK, such as gp130. While multiple activating receptors could bind a type of JAK, for instance, IL-2 and IL-4 both could activate JAK3. As is known to us, STAT is activated by JAK, while there is some selectivity in the activation of STAT by extracellular signaling factors.

Then, the subunits of receptors bind to JAK through the homologous ligand, and catalyze the tyrosine residues on the JAK structural domain. The catalyzed tyrosine residues in turn promote the phosphorylation of JAK and the formation of docking sites for surrounding amino acids. To add, GH induces the phosphorylation of JAK2, while IFN-α/β drives the phosphorylation of JAK1 and JAK2.

Activated JAK generates STAT binding sites, through the SH2 structural domain. The result is the subunit repositioning of STAT dimers, which leads to recruitment of STAT monomers to the binding site to assemble into dimers. Different extracellular signals produce corresponding STAT dimers, for instance, GH induces homodimers of STAT3 or STAT5, while IFN-α/β induces STAT1-STAT2 heterodimers, INF-γ induces STAT1-STAT3 heterodimers.^[50,55,56]^ Besides, STAT could also undergo covalent modifications, including phosphorylation, acetylation, and methylation of serine residues.^[15]^

**Figure 3.**
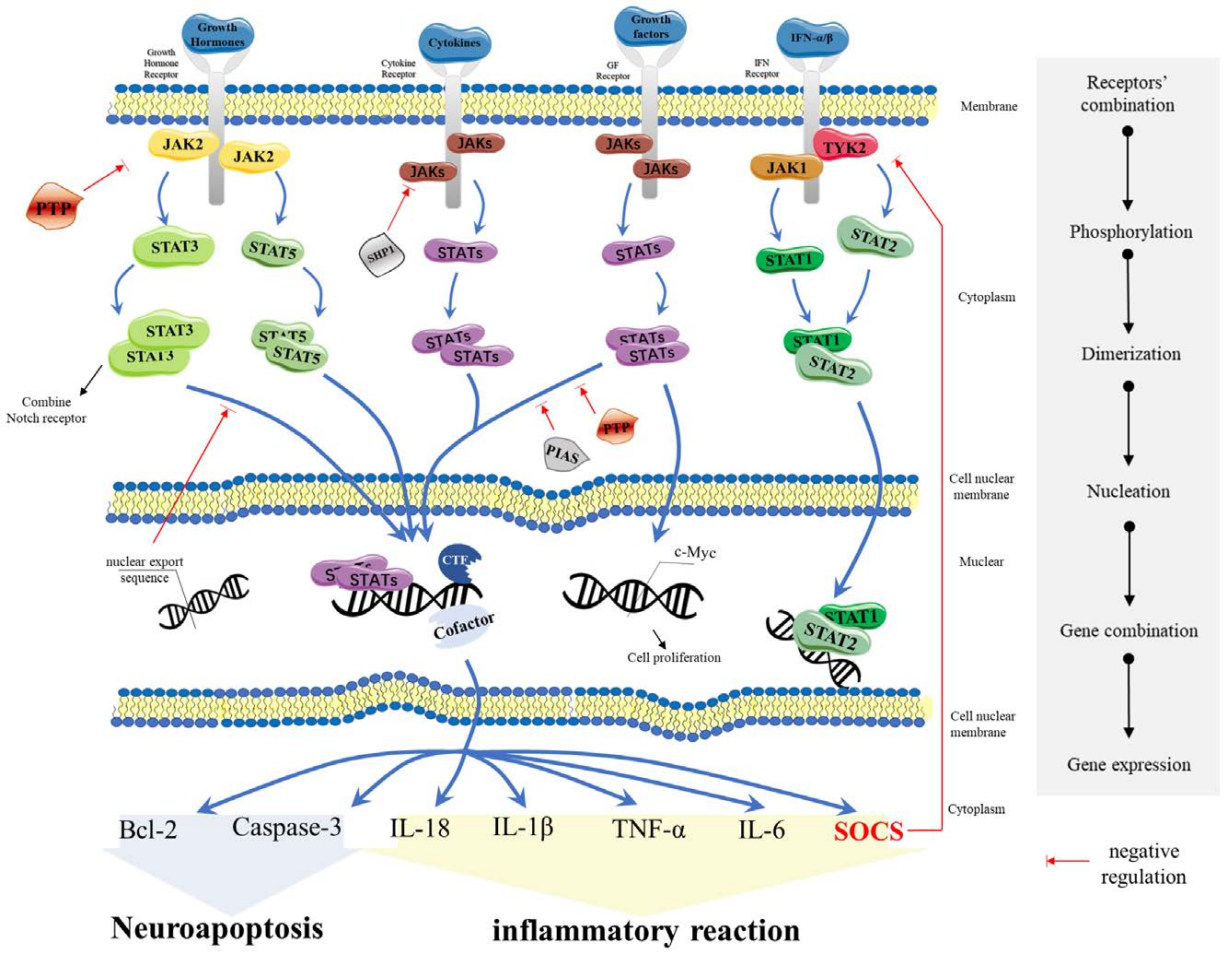
The JAK/STAT signaling pathway. JAK = Janus kinase, STAT = signal transducer and activator of transcription.

The STAT dimer enters the nucleus and binds to the regulatory sequence of the target gene or the promoter, up-regulating or down-regulating the expression of the DNA coding region. Then, the contents or activity levels of corresponding proteins changed at the time of translation, ultimately affecting cell proliferation, differentiation, and apoptosis. The specific process is shown in Figure 3.

Different dimers bind different kinds of components. Some upregulate the expression of Bcl-2 or caspase-3, regulating apoptosis. Others upregulate the expression of inflammatory factor genes, such as IL-18, exacerbating the inflammatory response.

#### 2.3.2. The negative regulation of the JAK/STAT signaling pathway.

The JAK/STAT signaling pathway is precisely regulated through negative feedback regulation. Negative feedback regulatory mechanisms include dephosphorylation, reduction of STAT binding activity to genes by PIAS, inhibition of signaling, and interference with STAT transport.^[15]^ It is mainly affected by the protein tyrosine phosphatase, PIAS and the suppressor of cytokine signaling (SOCS).^[14]^ Apart from this, the interference of nuclear transport, ubiquitin proteasome-mediated protein degradation, and dephosphorylation of STAT have also been shown in the research.^[57]^

##### 2.3.2.1. Dephosphorylation.

Dephosphorylation of JAK and STAT has an impact on STAT activation or reduces the amount of STAT dimers. This process is mainly mediated by protein tyrosine phosphatase, which could bind to complexes of p-JAK and receptor or directly to p-STAT to dephosphorylate the signaling molecule (Fig. 3).^[14]^

##### 2.3.2.2. The reduction of STAT-gene binding activity.

This process is mediated by PIAS. PIAS could bind to single STAT to affect dimerization, or bind directly to STAT dimers, or mask DNA binding regions.^[58]^ As is shown in Figure 3. Besides, the binding capacity of STAT and genes could be reduced by PIAS.^[14]^

##### 2.3.2.3. The inhibition of signal transduction.

It mainly involves feedback inhibition of SOCS.^[14]^ On the one hand, SOCS and STAT constitute a “feedback loop,” suggesting that p-STAT activates the expression of the SOCS gene. Of note, STAT is necessary for activation of SOCS.^[59]^ While SOCS counteracts STAT activation, as shown in Figure 3. On the other hand, SOCS could also bind to the receptor complex of p-JAK and block the pathway, favorable for anti-CIRI.^[60,61]^ It has also been shown that the kinase inhibitory region could inhibit JAK2 and block its activation.^[61]^ Overexpression of SOCS leads to apoptosis of neural stem cells.

##### 2.3.2.4. The interference with STAT transport

STAT transport is mainly composed of nuclear input and nuclear input The nuclear export sequence regulates the process of nuclear input, while the nuclear localization sequence regulates the process of nuclear output. When the pathway signal is attenuated, nuclear output is greater than nuclear input, decreasing the modulation expression intensity of STAT to the target DNA.^[15]^

## 3. The mechanism of CIRI based on JAK/STAT signaling pathway

According to the existing data, CIRI is caused by inflammation, oxidative stress, ferroptosis, pyroptosis, apoptosis, Ca^2+^ overload, and autophagy. These mechanisms are related to the JAK/STAT signaling pathway.^[62]^ Gene profiling studies in CIRI rats demonstrate the role of the JAK/STAT signaling pathway associated with the production of CIRI.^[26]^ Exploring the pharmacological effects of inhibitor AG490 of the pathway, researchers have revealed that they inhibit CIRI, suggesting that this pathway is a mechanism resulting in CIRI.^[63]^

### 3.1. The relationship between CIRI and neuroinflammation

#### 3.1.1. CIRI acts on inflammatory factors or cells to induce neuroinflammation.

The disruption of blood brain barrier integrity, brain edema and other symptoms occur in the ischemia reperfusion area.^[64]^ At this point, inflammatory factors are more likely to infiltrate tissues through the blood brain barrier, It leads to further destruction of brain tissue,^[65]^ and the absorption, metabolism and pharmacological effect of the drug.^[64]^ Moreover, microglia, astrocytes and NG2 glial cells are also activated in the ischemic area.^[66]^

Except for glial cells, inflammatory cells in the periphery also get activated, causing neuroinflammation. After the cerebral ischemia, the elevated expression levels of various adhesion molecules present on brain endothelial cells can be seen. The adhesion molecules consist of Vascular cell adhesion molecule-1, the intercellular cell adhesion molecule-1, P-selectin, and E-selectin. They could promote the flow of inflammatory immune cells into brain tissue.^[67]^

#### 3.1.2. CIRI increases inflammatory factors and cells via the JAK/STAT pathway.

Inflammatory factors exacerbate inflammatory damage or protect CIRI by acting on the JAK/STAT pathway. Under the condition of neuroinflammation after germinal matrix hemorrhage, IFN-α inhibits NF-κB via the JAK1/STAT1 signaling pathway to reduce neuroinflammation.^[68]^ Anti-IL-23 could activate the JAK2/STAT3 signaling pathway, causing the immune regulation in the development of CIRI.^[63]^ IL-22, involved in inflammatory responses, oxidative stress, and apoptotic, also activates STAT3 to improve ischemia-reperfusion death. Under the influence of IL-22, the phosphorylation of JAK2 and STAT3 is further increased to play a protective role.^[69]^ It has also been shown that IL-6 works in a dual way in CIRI,^[70]^ not only inducing an inflammatory response but also alleviating CIRI. Notably, it has been noted that activation of JAK3 after cerebral ischemia has no effect on the neuroinflammatory response.^[71]^

Instead of directly mediating neuroinflammation, the JAK/STAT pathway mediates the inflammatory response by transforming the microglia toward the M1 cell phenotype.^[72]^ There are many mechanisms of microglia recruitment in ischemically injured brain tissue. The Toll-like receptor,^[73]^ the Fas ligand, and other pattern recognition receptor are activated in microglia, that identify the damage-associated molecular pattern released by injured neurons.^[74]^ Therefore, the JAK/STAT pathway is activated, phosphorylating STAT, especially STAT3.^[51]^ Then p-STAT binds to on inflammation-associated genes,^[75]^ leading to the release of cytokines, including IL-1β, IL-6, IL-17, and IL-23.^[76]^ Specifically, the JAK2/STAT3 signaling pathway is activated, elevating the level of inflammatory vesicles.^[77]^ Also, STAT3 and p-STAT3 expression is seen in astrocytes in a brain ischemia/hypoxia model.^[51]^

It has been found that decreasing the phosphorylation of the JAK2/STAT signaling pathway inhibits the secretion of tumor necrosis factor-α (TNF-α) and IL-1β by microglia and then converses microglia to an anti-inflammatory state.^[75,78,79]^ In truth, glial cells have a highly activated response to JAK/STAT pathway, seen in the chronic central inflammation.^[79]^ For the immune regulation and inflammatory damage of microglia by mesenchymal stem cells, the JAK/STAT signaling pathway is also important.^[80]^ As is reported in a study, IFN-γ and IL-10 act on the JAK/STAT pathway and promote the expression of the B-cell activating factor in microglia to exert neuroprotective effects, demonstrating that microglia also played an active role in the inflammatory response.^[81]^

### 3.2. The relationship between JAK/STAT pathway and apoptosis

#### 3.2.1. CIRI mediates apoptosis through multiple mechanisms.

Apoptosis and necrosis are the major form of cell death in CIRI.^[82]^ After the occurrence of ischemia, 2 different modes of cell death as described above exist in different injury sites of brain tissue.^[83]^

Apoptosis is closely relevant to cellular inflammation, oxidative stress, and other regulatory mechanisms. Inflammation and oxidative stress generally promote apoptosis.^[16]^ Moreover, the process of apoptosis in the brain involves apoptotic signaling by mechanisms such as Ca^2+^ overload, caspase induction, excitatory amino acid toxicity, mitochondrial dysfunction, oxidative stress, immune inflammation, and so on.^[84]^

#### 3.2.2. CIRI also mediates apoptosis by the JAK/STAT pathway.

Except for mechanisms above, apoptosis is also linked to the JAK2/STAT3 signaling pathway.^[85]^ The JAK/STAT signaling pathway could be altered by some factors. There is experimental evidence that IL-21 binds to the receptor and affects apoptosis through the JAK/STAT pathway. IL-21-mediated apoptosis in turn promotes phosphorylation of STAT2, STAT5, and JAK.^[86]^

Some studies have demonstrated that apoptosis could be induced by the JAK2/STAT3 signaling pathway.^[83]^ When CIRI occurs, the phosphorylated JAK2/STAT3 signaling pathway increases the sensitized lipocalin2. Furthermore, lipocalin2 results in the proliferation of astrocytes and the release of inflammatory factors including IL-6, IL-18, TNF-α, and chemokines through neurons and some glial cells.^[87]^ The neuroinflammatory response takes place, further causing the death of relevant cells in the brain tissue. The JAK2/STAT3 pathway also induces apoptosis in neuronal cells by promoting the expression of caspase-2, 3, and 7 genes in the ischemic penumbra.^[83]^ Additionally, phosphorylation of STAT1 occurs in neurons within 24 hours of CIRI. p-STAT1 moves into the nucleus, and inhibits the PI3K/Akt pathway, altering the level of Bad and caspase3.

By contrast, STAT3 also has anti-apoptotic properties.^[88]^ In view of current clinical practice, drugs, hormones, receptor targeting or physical therapy could prevent apoptosis of neuronal cells by activating the signaling pathway with STAT3. Activated by JAK2/STAT3 pathway, STAT3 exerts the neuroprotective function. The function is associated with the Bcl-2 family and Bax.

### 3.3. The relationship between JAK/STAT pathway and cell proliferation and differentiation

#### 3.3.1. CIRI affects cell proliferation and differentiation through multiple mechanisms.

On the one hand, in the CIRI, the degree and direction of differentiation of the neural stem cells are affected. It is proven that the ROS system in mitochondria may be disturbed by cerebral ischemia, which enhances the oxidative response, induces neuronal apoptosis, and inhibits neuronal cell growth.^[89]^ Furthermore, Br-dU positive cell proliferation was triggered by CIRI, leading to proliferation of neural stem cells in the ventricular canal and subventricular zone on the ischemic side and differentiation towards astrocytes.^[90]^ The JAK/STAT pathway could also be activated by CIRI. It forms a crosstalk link with the Notch signaling pathway,^[91]^ and synergistically promotes the proliferation and differentiation of neural stem cells.

On the other hand, vascular proliferation, differentiation, and migration are affected. According to an experiment, the vascular endothelial growth factor (VEGF), basic fibroblast growth factor, the hypoxia-inducible factor, the angiopoietin and transforming growth factor are altered, after the onset of CIRI.^[3]^ Among them, VEGF and basic fibroblast growth factor function in the major way. More deeply, it has been demonstrated that upregulation the expressions of both promotes recovery from CIRI.^[92]^

#### 3.3.2. CIRI targets the JAK/STAT pathway to boost cell proliferation and differentiation.

In the development of CIRI, the underlying JAK/STAT pathway functions as a promoter of both proliferation and differentiation of glial cells, while an inhibitor of the production of neurons. There are more types of JAK/STAT pathways, while JAK2/STAT3 is major signaling pathway that impacts cell proliferation and differentiation. Therein, STAT3 is the main signaling molecule that maintains cell proliferation.

STAT3 enters the nucleus as a dimer and binds to DNA in the regulatory region of the target gene. Activated serine residues of STAT3 partially bind to Notch receptors.^[91]^ It boosts the expression of Hes3, which further promotes the expression of Sonic Hedgehog. The relevant research has suggested that the axonal injury led to STAT3 overexpression and neuronal reproduction. Whereas, lowering the level of STAT3 could transform neural stem cells to neurons and inhibit the ability of glial cell generation.^[93]^ After cerebral ischemia, estradiol, derived from astrocytes, could induce the JAK/STAT3 pathway. Thereby the proliferation of astrocytes is promoted, inhibiting the activation of microglia.^[93]^

Notably, phosphorylated JAK2 also promotes glial cell multiplication. STAT1-activated target genes act in opposite ways to STAT3 to promote inflammation and counteract proliferation.

Other receptors for erythropoietin, thrombopoietin and granulocyte-colony stimulating factor are also mediated by the JAK/STAT pathway. In particular, the proliferation and differentiation of erythrocytes, megakaryocytes and granulocytes are regulated by erythropoietin. It has been shown that histone deacetylase activates JAK1/STAT3 to promote angiogenesis, through inhibiting miR-19a.^[93]^

## 4. The role of the TCM for blood circulation and stasis removal in JAK/STAT pathway

Currently, studies have identified that TCM has positive effects on relieving, preventing CIRI.^[94]^ Meanwhile, the JAK/STAT pathway also has positive effects to alleviate CIRI. Does TCM work through the JAK/STAT pathway to prevent CIRI? Thus, this study mainly reviews TCM that act on JAK/STAT pathway.

Besides, through randomized controlled trials and the meta-analyses, blood-activating TCM combined with western medicine is reported to treat intracranial hemorrhage and ischemic stroke.^[95,96]^ Therefore, this study mainly reviews TCM with the effects of activating blood and resolving stasis and its ingredients.^[97]^

### 4.1. TCM herbal compound (Table 2)

#### 4.1.1. Naoluo Xintong Decoction.

Naoluo Xintong Decoction is composed of 6 herbs, including *Astragalus membranaceus* (Fisch.) Bge., *Panax notoginseng* (Burk.) F. H. Chen, *Ligusticum chuanxiong* Hort., *Angelica sinensis* (Oliv.) Diels, *Carthamus tinctorius* L., *Gastrodia elata* Bl., *Scolopendra subspinipes mutilans* L. Koch. Based on the pathogenesis of “Qi deficiency and blood stasis” in cerebral infarction recovery period, Naoluo Xintong Decoction has the effect of promoting blood circulation, removing blood stasis, tonifying qi, and strengthening energy.^[116]^

**Table 2:** Summary of TCM compounds.

TCM compounds	Herb composition		Effect	Treatment window time	Mechanisms related to the JAK/STAT pathway	References
Naoluo Xintong Decoction	*Astragalus membranaceus* (Fisch.) Bge.	30 g	Promoting blood circulation, removing blood stasis, tonifying qi, and strengthening energy	No reports	Promoting the expression of SOCS mRNA to enhance the negative feedback regulation of JAK/STAT pathway Inhibiting prolactin receptor protein expression and the JAK/STAT pathway to counteract apoptosis and promote differentiation of neural stem cells	^[68,98–100]^
	*Ligusticum chuanxiong* Hort.	10 g				
	*Panax notoginseng* (Burk.) F. H. Chen	6 g				
	*Angelica sinensis* (Oliv.) Diels	10 g				
	*Carthamus tinctorius* L.	10 g				
	*Gastrodia elata* Bl.	10 g				
	*Scolopendra subspinipes mutilans* L. Koch	4 g				
Buyang Huanwu Decoction	*Astragalus membranaceus* (Fisch.) Bge.	120 g	Used in the treatment of stroke and restoration of neurological dysfunction	Within 4h of CIRI (dosage 40 g/kg)	Inhibiting p-JAK2 and p-STAT3 to decrease Bax and counteract apoptosis Inhibiting the JAK/STA3 signalling pathway to reduce proliferation of vascular smooth muscle	^[99–112]^
	*Angelica sinensis* (Oliv.) Diels	6 g				
	*Ligusticum chuanxiong* Hort.	3 g				
	*Paeonia lactiflora* Pall.	4.5 g				
	*Prunus persica* (L.) Batsch	3 g				
	*Carthamus tinctorius* L.	3 g				
	*Pheretima aspergillum* (E.Perrier)	3 g				
Huoxue Rongluo Decoction	*Spatholobus suberectus* Dunn	0.6 g	Based on the mechanism of “Yin deficiency, blood stasis, grouping of ligaments-Rong Qi deficiency and stagnation” Nourishing yin and the blood	No reports	Up-regulating JAK2 and STAT3 to increase mitochondrial autophagy and promote angiogenesis	^[113–115]^
	*Piper puberulum* (Benth) Maxim.	0.6 g				
	*Rehmannia glutinosa* Libosch.	0.3 g				
	*Scrophularia ningpoensis* Hemsl	0.2 g				
	*Polygonatum sibiricum* Red.	0.3 g				
	*Boswellia carterii* Birdw.	0.2 g				
	*Commiphora myrrha* Engl.	0.2 g				
	*Ligusticum chuanxiong* Hort.	0.2 g				

JAK = Janus kinase, SOCS = suppressor of cytokine signalling, STAT = signal transducer and activator of transcription, TCM = traditional Chinese medicine.

Naoluo Xintong Decoction could inhibit TNF-α, affecting the plasticity and functional recovery of nerves. It not only alleviates inflammation, but promote the proliferation of endothelial cells (L Wang et al, 2018). It could also reduce the expression of caspase-12 within 24 hours of CIRI, ultimately improve CIRI.^[117]^

For the effects on cell proliferation and differentiation, Naoluo Xintong Decoction not only facilitates the mRNA expression of SOCS5, but also restrains the level of prolactin receptor and the JAK/STAT pathway. Thus, Naoluo Xintong Decoction promotes the differentiation of neural stem cells into neurons and their migration.^[98]^ The process reduces the degree of neuronal apoptosis and facilitates the recovery of CNS function after CIRI. Additionally, Naoluo Xintong Decoction can increase VEGF, improve microvascular density, reduce apoptosis of endothelial cells in the semidark zone, and produce vasoprotective effects.^[68]^ Few clinical trials study the function of Naoluo Xintong Decoction.

#### 4.1.2. Buyang Huanwu Decoction.

Buyang Huanwu Decoction is composed of *Astragalus membranaceus* (Fisch.) Bge.*, Paeonia lactiflora* Pall., *Pheretima aspergillum* (E.Perrier), *Angelica sinensis* (Oliv.) Diels, *Ligusticum chuanxiong* Hort., *Carthamus tinctorius* L., *Prunus persica* (L.) Batsch. It is used in the treatment of stroke and restoration of neurological dysfunction.^[99,100]^ A double-blind, randomized controlled trial has proved that Buyang Huanwu Decoction could reduce the nerve damage.^[101]^

Buyang Huanwu Decoction affects the inflammatory response, protecting the nerves, and fighting apoptosis.^[102]^ It decreases TNF-α and inflammatory cells in the ischemic penumbra, inducing the differentiation potential of neural stem cells to neurons.^[103]^ Additionally, Buyang Huanwu Decoction improves the plasticity of neuronal synapses,^[104]^ and upregulates the expression of VEGF, stimulating the cell proliferation.^[105]^

Buyang Huanwu Decoction could down-regulate the expression of p-JAK2 as well as p-STAT3, suppress the JAK2/STAT3 pathway, decrease Bax expression, diminish the number of neuronal apoptosis, and attenuate neurological deficits.^[106,107]^ Oppositely, Buyang Huanwu Decoction significantly activates the p-PI3K/Akt/-Bad and JAK2/STAT3/cell cycle protein D1 signaling pathways in vitro *and* in vivo, exerting neuroprotective effects.^[108]^ Notably, based on a study on the components of glycoside in Buyang Huanwu Decoction, it has been proved to inhibit the JAK/STAT3 pathway, suppressing the proliferation of vascular smooth muscle cells.^[109]^

According to the network pharmacology results, *Hedysarum multijugum Maxim.*-*Chuanxiong rhizoma* compound, a herbal formula modified from Buyang Huanwu decoction, could act on the JAK/STAT signaling pathway to alleviate inflammation.^[110]^ Several clinical trials have suggested that Buyang Huanwu Decoction alleviates ischemic stroke of patients through improving hemorheologic indexes.^[111]^ Besides, Buyang Huanwu Decoction, combining with Photosensitized Oxidation Auto-Hemotherapy, could improve the stroke sequela.^[112]^ It may also help to improve the post-stroke fatigue.^[100]^

#### 4.1.3. Huoxue Rongluo Decoction.

The formula is composed of *Spatholobus suberectus* Dunn, *Piper puberulum* (Benth) Maxim., *Rehmannia glutinosa* Libosch., *Scrophularia ningpoensis* Hemsl, *Polygonatum sibiricum* Red., *Boswellia carterii* Birdw., *Commiphora myrrha* Engl., *Ligusticum chuanxiong* Hort. This formula has the effect of nourishing yin and the blood.^[113]^

The study have showed that Huoxue Rongluo Decoction alleviates CIRI by down-regulating miR-370-3p, activating the expression of JAK2/STAT3 pathway, increasing downstream VEGF, enhancing microvascular density in the ischemic area, and improving the degree of cerebral vascular neovascularization.^[114,115]^

In addition to the TCM herbal compound mentioned above, studies have shown that Sijunzi Decoction and Yupingfeng Powder could also regulating the level of JAK and STAT to influence the expression of inflammatory factors including IL-4.^[118]^

### 4.2. TCM herb pairs (Table 3)

TCM herb pairs are made by combining 2 herbs and play a major role in Chinese herbal medicine compounding. Observing the above herbal compound, we realize that *Angelica sinensis* (Oliv.) Diels*-Ligusticum chuanxiong* Hort. pair, *Astragalus membranaceus* (Fisch.) Bge.*-Panax notoginseng* (Burk.) F. H. Chen pair appears several times, and some studies shows that they could alleviate CIRI.

**Table 3:** Summary of TCM herb pairs.

TCM herb pairs	Composition	Effect	Active ingredients	Mechanisms related to the JAK/STAT pathway	References
*Angelica sinensis* (Oliv.) Diels*-Ligusticum chuanxiong* Hort. Pair	*Angelica sinensis* (Oliv.) Diels	Nourishing blood, invigorating the blood circulation, regulating the menstruation, and relieving the pain	Astragalus polysaccharide	Decreasing the JAK2 in the ischemic penumbra and p-STAT3 in the cell nucleus, to reduce cyclooxygenase-2 and intercellular cell adhesion molecule and counteract neuroinflammation	^[119–122]^
	*Ligusticum chuanxiong* Hort.	Moving qi, dispersing blood, dispeling “wind,” and relieving pain	Ligustrazine		
*Astragalus membranaceus* (Fisch.) Bge.-*Panax notoginseng* (Burk.) F. H. Chen	*Astragalus membranaceus* (Fisch.) Bge.	Nourishing the blood, promoting stagnation, stimulating paralysis, and eliminating toxin and pus	Astragaloside	Inhibiting the expression of p-JAK1, p-STAT1, p-JAK2, STAT3, p-STAT3, to counteract apoptosis and improve the blood-brain barrier. Improving energy metabolism, act on some inflammatory factors and promote the recovery of nerve function	^[123–138]^
	*Panax notoginseng* (Burk.) F. H. Chen	Stopping bleeding, dissipating blood stasis, and relieving pain. Related to inhibition of apoptosis, inflammatory response, and neuron protection	*Panax notoginseng* saponins		

JAK = Janus kinase, STAT = signal transducer and activator of transcription, TCM = traditional Chinese medicine.

#### 4.2.1. *Angelica sinensis* (Oliv.) Diels-*Ligusticum chuanxiong* Hort. pair.

*Angelica sinensis* (Oliv.) Diels, known as Dang Gui in Chinese, whose main functions are to nourish blood, invigorate the blood circulation, regulate the menstruation, and relieve the pain. It could also protect brain tissue through promoting angiogenesis or inhibiting inflammatory response and apoptosis. One of its important active ingredients for blood activation and anti-inflammation is *Angelica* polysaccharide.^[119]^

*Ligusticum chuanxiong* Hort., an herb known as Chuan Xiong in Chinese, whose main functions are to move qi and disperse blood, dispel “wind,” and relieve pain. Ligustrazine, one of its main components, also has anti-cerebral ischemic effect.^[120]^ Ligustrazine improves the blood-brain barrier in CIRI through the JAK/STAT signaling pathway.^[121]^ It has been reported that different dosage of ligustrazine could regulate the hemorrheologic parameters and fibrinogen.^[120]^

Related studies have demonstrated that *Angelica sinensis* (Oliv.) Diels*-Ligusticum chuanxiong* Hort. pair, as a classical blood activator and blood stasis remedy pair, has the effect of tonifying and activating blood. It is mostly used in the Ying blood stasis syndrome.^[121,122]^ It reduces the level of JAK2 in the cytoplasm of the ischemic penumbra and p-STAT3 in the nucleus. Besides, it inhibits the JAK/STAT pathway, and decreases the transcription and translation of inflammatory factors such as cyclooxygenase-2 and intercellular cell adhesion molecule, to achieve a reduction in cerebral infarct size and improve edema.^[121,122]^

#### 4.2.2. *Astragalus membranaceus* (Fisch.) Bge.-*Panax notoginseng* (Burk.) F. H. Chen pair.

*Astragalus membranaceus* (Fisch.) Bge., known as Huang Qi in Chinese, has the effect of nourishing the blood, promoting stagnation, stimulating paralysis, and eliminating toxin and pus. Its active ingredient is astragaloside, with anti-inflammatory activity and anti-oxidant activity.^[123]^

*Panax notoginseng* (Burk.) F. H. Chen, known as San Qi in Chinese, is useful for stopping bleeding, dissipating blood stasis, and relieving pain. It often used to treat hemoptysis, epistaxis, blood in stool, pain and swelling in the clinical practice.^[124]^ *Panax notoginseng* saponins (PNS) is the active ingredients, containing ginsenoside Rg1, ginsenoside Rb1, notoginseng R1 and so on.^[125]^ The saponin is known to improve blood circulation, dispel blood stasis, and smooth blood vessels.^[126,127]^ It is related to inhibition of apoptosis, inflammatory response, and neuron protection.^[128,129]^ It has been reported that PNS has the effect of neuron protection,^[128]^ and the extract of *Panax notoginseng* (Burk.) F. H. Chen could improve the ischemic stroke in a safe way.^[130]^

*Astragalus membranaceus* (Fisch.) Bge*.-Panax notoginseng* (Burk.) F. H. Chen pairs are commonly used to supplement Qi and activate blood circulation. A current clinical report has shown that *Astragalus membranaceus* (Fisch.) Bge.*-Panax notoginseng* (Burk.) F. H. Chen pairs decrease the level of matrix metalloproteinases (MMPs) to treat type 2 diabetic macroangiopathy.^[131]^ Researches have suggested that Astragalus extracts and Panax notoginseng saponins could reduce apoptosis, improve energy metabolism, and act on some inflammatory factors to alleviate CIRI.^[132,133]^ Furthermore, astragaloside, in combination with PNS, effectively prevents the phosphorylation of JAK1 and STAT1, and reduces apoptosis.^[134,135]^ Using a network pharmacology, a study has revealed that Xueshuantong, consisting of PNS, lowers the level of proteins in the signaling pathway, including STAT3 and p-STAT3, and improves the extent of blood-brain barrier disruption.^[136]^ Xueshuantong could also promote the recovery of nerve function, as a clinical research reported.^[137]^ A clinical study has demonstrated that Xue Shuan Tong could have an impact on carotid intima-media thickness to improve patients with cerebral infarction.^[138]^

### 4.3. TCM ingredients (Table 4)

Apart from the above-mentioned herbal compounds and pairs, active ingredients extracted from plants, such as *Carthamus tinctorius* L., *Polygonum cuspidatum* Sieb. et Zucc. and *Pueraria lobata* (Willd.) Ohwi, have been verified to promote blood circulation and resolve blood stasis. Besides, it could also act on the JAK/STAT signaling pathway.

**Table 4:** Summary of TCM ingredients.

TCM ingredients	Herb extracted from	Mechanisms related to the JAK/STAT pathway	References
Hydroxysafflor yellow A	*Carthamus tinctorius* L.	Decreasing p-JAK2, p-STAT5 to facilitate negative feedback regulation of SOCS and counteract neuroinflammation. Decreasing the levels of JAK2 and STAT3	^[59,139–141]^
Resveratrol	*Polygonum cuspidatum* Sieb. et Zucc.	Inhibiting the JAK/STAT1 signaling pathway and decreasing STAT1, JAK2, p-JAK2, p-STAT3, to alleviate inflammation and regulate the level of Bcl-2 and Bax. Inhibiting the JAK1/STAT3 signaling pathway to promote recovery of neural function. Conversely, hyperactivating the JAK2/STAT3 pathway or increasing p-JAK1, p-STAT3, to promote negative feedback of SOCS and reduce inflammation	^[72,142–149]^
Puerarin	*Pueraria lobata* (Willd.) Ohwi	Decreasing JAK2, STAT3, p-STAT3 in the ischemic penumbra to reduce apoptosis. Conversely, up-regulating JAK2, STAT3 to protect neurons	^[150–153]^
Formononetin	*Astragalus membranaceus* (Fisch.) Bge.	inhibit the relevant targets on JAK2/STAT3 signaling pathway to play a role of anti-inflammatory and anti-apoptotic	^[154]^

JAK = Janus kinase, SOCS = suppressor of cytokine signaling, STAT = signal transducer and activator of transcription, TCM = traditional Chinese medicine.

#### 4.3.1. Hydroxysafflor yellow A.

Hydroxysafflor yellow A (HSYA) is a kind of safflower yellow. Safflower yellow, composed of various chalcones, is one of the main active ingredients of *Carthamus tinctorius* L. It exerts multiple protective effects in cerebrovascular disease.^[155]^ *Carthamus tinctorius* L., known as Hong Hua in Chinese, has the function of activating blood circulation, dispersing blood stasis, and relieving pain. It is used for the dysmenorrhea, the abdominal pain due to blood stasis, and bruising and the traumatic injury. Moreover, it is one of the important components of compound angelica injection. Compound angelica injection downregulates the JAK2/STAT3 signaling pathway to reduce the release of inflammatory factors, is likely associated with *Carthamus tinctorius* L. Further speaking, the function of *Carthamus tinctorius* L. in activating blood circulation and resolving blood stasis is mainly associated with HAYA.^[155]^ Safflower yellow has also reported to regulate the expression of hepcidin by downregulating the JAK/STAT3 pathway, to decrease the Iron Overload.^[156]^

A clinical trial has shown that HSYA has therapeutic effect on ischemic stroke with certain efficacy and safety.^[157]^ HSYA mitigated myocardial ischemia/reperfusion injury by inhibiting the activation of the JAK2/STAT1 pathway.^[139]^ It has been noted that HSYA regulates the underlying JAK2/STAT3 signal transduction pathway and promotes SOCS3 expression to treat CIRI.^[59]^ Meanwhile, Panlongqi tablet, containing HSYA may regulate the JAK/STAT pathway to decrease the levels of IL-1β, IL-6, IL-17, improving adjuvant-induced rheumatoid arthritis.^[140]^ HSYA has also reported to promote neuroprotection partially through JAK2/STAT3 pathway.^[141]^

#### 4.3.2. Resveratrol.

Resveratrol (RV), a non-flavonoid polyphenolic compound, is present in the *Polygonum cuspidatum* Sieb. et Zucc. *Polygonum cuspidatum* Sieb. et Zucc., known as Hu Zhang in Chinese, is effective in activating blood circulation, dispersing blood stasis, promoting menstruation, and relieving cough. Clinically, it widely used in the arthralgia, traumatic injury, cough, excessive phlegm, etc. Its extract, RV, has a wide range of antioxidant properties, with anti-inflammatory, anti-cancer, and cardiovascular protective effects.^[158,159]^

The study has proven that RV is used in inflammatory diseases and is helpful in relieving neuroimmune dysfunction. It blocks the JAK-STAT1 pathway and control the inflammatory response of IFN-γ-activated macrophages. It can also inhibit the activation of JAK2.^[14]^ Additionally, by inhibiting STAT1, RV decreases rotenone-induced neuroinflammation in microglia.^[160]^ RV also increases M2-type anti-inflammatory microglia and suppresses inflammatory responses, through activating STAT3 and STAT6.^[161]^

In view of a research, RV decrease the phosphorylation of JAK, STAT, extracellular regulatory kinase or c-Jun N-terminal kinase. Also, it could inhibit the JAK/ERK/STAT signaling pathway and regulate the levels of apoptotic proteins, containing Bcl-2 and Bax.^[142]^ Ultimately, it protects the neurons in the hippocampal region from CIRI. There are also studies that have identified that the neuroprotective effect of RV is based on the inhibition of macrophages and microglial.^[143]^ While some studies have identified that RV could improve astrocyte survival.^[144,145]^ It has been suggested that resveratrol down-regulates the JAK1/STAT3 signaling pathway, allowing the restoration of neuroimmune function.^[146]^ RV also targets on the TLR4/NF-Kb/STAT3 pathway to inducing the neuroinflammation.^[147]^

However, RV is not the only inhibitor of JAK/STAT. According to a study, RV is observed to exert neuroprotective effects by indirectly upregulating the PI3K/AKT/mTOR signaling pathway through activation of the JAK2/STAT3 signaling pathway.^[148]^ RV also upregulates IL-10 and directly increases p-JAK1 and p-STAT3.^[72]^ High level of p-STAT3 increases the expression of SOCS, negatively feeding back on inflammation-related signaling pathways and inhibiting the activity of microglia.^[149]^

#### 4.3.3. Puerarin.

Puerarin (PUE) is an isoflavone derivative isolated from *Pueraria lobata* (Willd.) Ohwi, a member of the legume family.^[162]^ *Pueraria lobata* (Willd.) Ohwi, known as Ge Gen in Chinese, could reduce the fever, eliminate the rash, promote the production of fluids, quench thirst, and replenish yang to stop diarrhea. One of the main components of its function is PUE. The research has identified that PUE increases microvascular density^[158]^ and fights inflammation,^[163]^ and is commonly used clinically for coronary angina and hypertension. PUE combined with naloxone has clinical efficacy in the treatment of traumatic cerebral infarction.^[163]^

Several studies have demonstrated that PUE significantly reduces JAK2 and STAT3 levels in the infarcted region.^[150]^ Moreover, studying the various time points of PUE to alleviate CIRI, it has been found that the longer the treatment time of PUE, the stronger the inhibition of JAK2 and STAT3 expression.^[151]^ It has been certificated that PUE could modulate the erythropoietin receptor-JAK2-STAT5 signaling pathway to alleviate the extent of brain injury, and downregulate erythropoietin levels. The process decreases the phosphorylation level of JAK2, which ultimately reduces the level of the downstream signaling factor STAT5.^[152]^ PUE down-regulates proteins involved in the olfactory transduction pathway and JAK2/STAT3 pathway, increasing the activity of astrocyte and exerting a protective effect.^[150]^

Yet, in contrast, PUE could also trigger the catalytic activity of intracellular structural domains by activating more α7 nicotinic acetylcholine-like receptors, recruiting and phosphorylating JAK2. And eventually, the expression of STAT3 mRNA is improved.^[153]^

#### 4.3.4. Formononetin.

Formononetin (FMN) is a phytoestrogen member of the flavonoid family and is found in legumes and several types of clovers like *Astragalus membranaceus* (Fisch.) Bge.^[150]^ *Astragalus membranaceus* (Fisch.) Bge. is known as Huang Qi in Chinese, and the research have demonstrated that FMN has the function of anti-inflammatory, antioxidant, and anti-allergic.^[164]^ Based on some studies, it is proven effective in the treatment of hepatocellular carcinoma,^[165]^ senile cognitive impairment,^[166]^ triple negative breast cancer,^[167]^ and neurological diseases.^[168]^

Several studies have proven that FMN is effective in treating CIRI. In view of a research, FMN can decrease endoplasmic reticulum stress-mediated apoptosis,^[169]^ promote vascular endothelial growth factors,^[170]^ and promote neuroprotective.^[17]^ FMN also activates the PI3K/Akt signaling pathway and decrease the Bax/Bcl-2 ratio to improve the neurological deficit in CIRI.^[171]^

Based on the rat study, FMN can inhibit the JAK2/STAT3 signaling pathway to decrease inflammatory factors or apoptosis-related factors to relieve CIRI.^[154]^ However, there is still a lack of clinical researches to prove that FMN plays a role in treating CIRI through the JAK/STAT signaling pathway.

## 5. Problems and prospects

Reperfusion injury is a greater risk factor for cerebral ischemic, and its pathogenesis is regulated by multiple cellular pathways, of which the JAK/STAT pathway is one. The JAK/STAT pathway plays a predominantly negative regulatory role in CIRI. On the one hand, the JAK/STAT pathway mediates the production of inflammatory factors or recruitment of inflammatory cells by microglia, while the JAK/STAT pathway increases the concentration of lipocalin2, glial cells and mediates neuronal apoptosis. On the other hand, the presence of related proteins in the STAT family could have a direct effect on cell proliferation and differentiation. Nevertheless, some studies have demonstrated that the JAK/STAT pathway has a neuroprotective effect. Thereby, the corresponding mechanism needs to be further investigated and explored.

Currently, TCM acting on the JAK/STAT pathway is extensively studied. However, the specific mechanisms remain unclear. Some inhibit the JAK/STAT pathway, while others promote it. For TCM that activates blood circulation and remove blood stasis, there is still much to be explored. Some TCM have clear anti-CIRI efficacy without clear indication of action on JAK/STAT signaling pathway. While others have been reported in ischemia/reperfusion injury in other organs through the JAK/STAT signaling pathway, yet there are few reports on neurology. Meanwhile, the researches on the molecular mechanism of TCM in treating CIRI mainly stay in preclinical researches, and there are few clinical studies. Thus we still need further clinical researches to confirm the function of JAK/STAT signaling pathway in TCM treating CIRI. Besides, considering that the safety and clinical indications of the TCM are still unclear, higher quality clinical trials are severely needed. The TCM herbal compounds and drug pairs have multi-target characteristics, which need to be clarified by clinical research as well.

## Author contributions

**Formal analysis:** Tianzhi Song, Yishu Zhang, Liangrong Zhu.

**Visualization:** Tianzhi Song.

**Writing – original draft:** Tianzhi Song, Yuyan Zhang.

**Writing – review & editing:** Tianzhi Song, Yuyan Zhang, Jingmei Song.
